# Common Neural Mechanisms Underlying Reversal Learning by Reward and Punishment

**DOI:** 10.1371/journal.pone.0082169

**Published:** 2013-12-11

**Authors:** Gui Xue, Feng Xue, Vita Droutman, Zhong-Lin Lu, Antoine Bechara, Stephen Read

**Affiliations:** 1 National Key Laboratory of Cognitive Neuroscience and Learning and IDG/McGovern Institute for Brain Research, Beijing Normal University, Beijing, China; 2 Department of Psychology, University of Southern California, Los Angeles, California, United States of America; 3 Center for Cognitive and Behavioral Brain Imaging and Department of Psychology, The Ohio State University, Columbus, Ohio, United States of America; 4 Brain and Creativity Institute, University of Southern California, Los Angeles, California, United States of America; Duke University Medical Center, United States of America

## Abstract

Impairments in flexible goal-directed decisions, often examined by reversal learning, are associated with behavioral abnormalities characterized by impulsiveness and disinhibition. Although the lateral orbital frontal cortex (OFC) has been consistently implicated in reversal learning, it is still unclear whether this region is involved in negative feedback processing, behavioral control, or both, and whether reward and punishment might have different effects on lateral OFC involvement. Using a relatively large sample (N = 47), and a categorical learning task with either monetary reward or moderate electric shock as feedback, we found overlapping activations in the right lateral OFC (and adjacent insula) for reward and punishment reversal learning when comparing correct reversal trials with correct acquisition trials, whereas we found overlapping activations in the right dorsolateral prefrontal cortex (DLPFC) when negative feedback signaled contingency change. The right lateral OFC and DLPFC also showed greater sensitivity to punishment than did their left homologues, indicating an asymmetry in how punishment is processed. We propose that the right lateral OFC and anterior insula are important for transforming affective feedback to behavioral adjustment, whereas the right DLPFC is involved in higher level attention control. These results provide insight into the neural mechanisms of reversal learning and behavioral flexibility, which can be leveraged to understand risky behaviors among vulnerable populations.

## Introduction

Adaptive behaviors require the ability to quickly adjust responses in changing environments. This behavioral flexibility is often measured using a reversal learning paradigm, in which participants need to effectively overcome established associations and learn new ones based on feedback. Impairments in reversal learning are associated with a wide range of behavioral abnormalities or psychiatric conditions characterized by impulsiveness and disinhibition [1], [2], [3], [4], [5], such as reactive aggression [6], psychopathy [7], [8], Obsessive compulsive-disorder [9], [10], severe conduct disorder [11], and bipolar disorder [12].

Reversal learning is a complex task that involves many components. Understanding its neural mechanisms is further complicated by the use of different tasks across studies. First used on animals [13], [14], the classic reversal learning task uses a preference reversal paradigm, in which one of the two stimuli is rewarded and the contingency is reversed at a certain point. Subjects are asked to choose the correct stimulus and reverse their preference when the contingency is changed. Lesion studies on animals [3], [13], [14], [15], [16] and humans [2], [17] have consistently implicated the ventrolateral prefrontal cortex and lateral orbitofrontal cortex (OFC) in this type of reversal learning. Mirroring these findings, functional imaging studies have also identified the lateral OFC [9], [18], [19], and several other brain regions in reversal learning, including the inferior frontal gyrus (IFG) [20], [21], the dorsomedial prefrontal cortex (DMPFC)[22], [23], the dorsolateral prefrontal cortex (DLPFC) [23], [24], the posterior parietal cortex [25], [26], and the striatum [20], [27], [28], [29], [30], [31].

What is less frequently examined is how reward and punishment modulate reversal learning. Reward and punishment represent two major motivations to learn in changing environments. Focusing on reinforcement learning, convergent evidence from patients, pharmacological and functional imaging studies has revealed distinct mechanisms underlying learning from positive and negative feedback. For example, unmedicated Parkinson Disease (PD) patients with low striatal dopamine were better at learning from punishment relative to reward [32], [33], whereas medicated PD patients [32], [33] or healthy subjects [34] with high baseline dopamine levels in the striatum were better at learning from reward than punishment. Using a modified version of the probabilistic learning task developed by Frank *et al.* [33], Wheeler & Fellows [35] found that the ventromedial prefrontal cortex (VMPFC) was specifically involved in learning by negative feedback. A recent lesion study also suggests that patients with damage in the anterior insula and dorsal striatum were specifically impaired in punishment-based avoidance learning [36]. Using fMRI, it has been found that the posterior dorsal striatum responded only to unexpected reward, whereas the anterior ventral striatum responded to both unexpected reward and unexpected punishment [37]. Similar dissociations between positive and negative prediction errors have been observed in the striatum [38], [39] and in the striatum and amygdala [40].

It is unclear whether similar dissociations between reward and punishment could be found for reversal learning. Two methodological issues must be considered in examining the effect of reward and punishment feedback on reversal learning. In the serial reversal paradigm used by many studies, the reversal of contingency was almost always signaled by a negative feedback. It is thus difficult to tell whether the lateral OFC is involved in negative feedback processing [19] or inhibition *per se*. Moreover, these studies did not focus on contrasting reversal learning with initial acquisition or general reinforcement learning [22], [41]. To address this issue, several studies have tried to either compare reversal errors with probabilistic errors and nonreversal errors in a serial reversal task [24], or compare reversal errors with acquisition errors [22], [41]. Ghahremani et al. [41] directly compared the first reversal errors with the first incorrect acquisition trials, and the first correct post-reversal trials with the 2^nd^ correct acquisition trials. They found common activations in the lateral OFC for both contrasts and additional activations in the right DLPFC and caudate for the first contrast. Hampshire et al. [42] compared the switching events during acquisition and reversal and found particularly strong activations in the lateral OFC for reversal, whereas the LPFC showed equivalent activations in both conditions. To examine the expression of new behavior under extended interference, Xue et al. [43] examined the reversal of extensively trained associations and found activations in the ACC-IFG-PPC network several repetitions after reversal, suggesting their role in expressing new behavior under the interference of strong old associations.

The second issue concerns the kind of positive and negative feedback used. Monetary gain and loss were commonly used as positive and negative feedback in previous studies. The effectiveness of this type of negative feedback might be complicated by the ethical consideration that subjects should never lose money [35], [39]. Still, although overlapping neural mechanisms for reward and reward prediction errors have been identified for primary and secondary rewards using money and juice [44], [45], it is not clear whether monetary loss is analogous to a primary punishment, such as air puff or electric shock in driving behavioral flexibility.

The present study aimed at parsing the subprocesses (i.e., detecting contingency change vs. expression of new behavior by inhibiting old association) associated with reversal learning, and further examined how they were modulated by reward and punishment. To that end, we used a deterministic learning paradigm modified from Ghahremani et al. [41], in which subjects were asked to learn the association (with 5 to 8 repetitions) between a novel image and a left or right key press through deterministic feedback. The contingency was then reversed and subjects learned the new contingency over 5 repetitions to achieve high accuracy. Unlike the serial reversal learning paradigm, the contingency for each image was reversed only once and phased out after the predetermined number of repetition had been reached, and new images were then introduced. Under the reward condition, subjects received one point (convertible to real money at the end of the experiment) for each correct response but otherwise nothing; under the punishment condition, subjects received a mild electric shock for each incorrect response but otherwise nothing. By comparing the brain responses at various stages of learning between initial acquisition and reversal, we could clearly dissociate the subprocesses specific to reversal learning. Furthermore, the feedback manipulation could help elucidate the role of reward and punishment in modulating these processes.

This study is also a part of a large-scale project examining the behavioral and neural mechanisms of risky decision-making among men who has sex with men (MSM). Although only 4% of the US population, MSM constitute half of all new cases of HIV. Our study thus represents the first step to understand the neural mechanism of cognitive control among this population, which can be leveraged to understand their risky behaviors.

## Materials and Methods

### Ethics statement

The experiment was conducted in compliance with the Code of Ethics of the World Medical Association (Declaration of Helsinki) and the protocol of the fMRI study was approved by the Institutional Review Board at the University of Southern California.

### Participants

Forty-seven male subjects (15 Caucasians, 19 black and 13 Hispanic. Age: 19 to 31 years old; mean = 25.36 years) participated in the experiment. They qualified for this study if they were non-binge drinkers, HIV negative (tested within last 6 months), free of neurological or psychiatric history, and met all safety requirements for MRI scan. Informed written consent was obtained from each subject before the experiment.

### The deterministic reversal learning task

**Figure 1:**
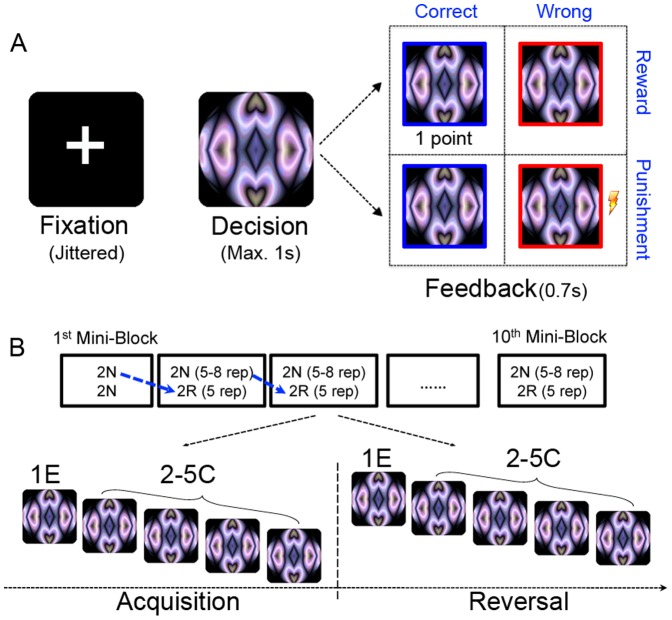
Experimental design. (A) Trial structure and feedback schedule. Participants were presented with an abstract image and had up to 1 s to make a category judgment (left or right key). Under the reward condition, they received 1 point for a correct response but otherwise nothing; under the punishment condition, they received a moderate shock for each wrong response but otherwise nothing. Under both conditions, they also received information feedback (blue frame for correct responses and red frame for wrong responses). The feedback lasted for 0.7 s, which was followed by a fixation cross for an average ISI of 3 s (taken from an exponential distribution ranging from 2.5 to 7.8 s). (B) Reversal learning paradigm. Trials were presented in mini-blocks of 4 images (two new images as acquisition trials and two old images from the last block as reversal trials) that were repeated 5 times. To prevent subjects from being able to predict reversals, the new images might be repeated an additional 0,1, 2 or 3 times before the contingency was reversed. The images were then phased out of the experiment after 5 post-reversal repetitions. We compared the first error (1E) between reversal and acquisition to examine the neural regions involved in the detection of contingency change. In contrast, we compared the correct trials during repetition 2 to 5 (2–5C) between reversal and acquisition to examine the neural regions involved in the expression of new behaviors under the interference of old behaviors.

Figure 1 depicts the stimuli and the deterministic reversal learning task, which was modified from Ghahremani et al. [41]. In this task, subjects were presented with an abstract computer-generated visual pattern (ArtMatic Pro, U&I Software LLC, http://uisoftware.com) and asked to decide whether it was associated with a left or right key response, via trial and error. The picture was presented for 1 s, during which participants made their response. After the response, feedback was delivered according to subjects' response and the experimental conditions. Under the reward condition, subjects would receive one point for each correct response but nothing for each incorrect response. The point was later converted to dollars at a ratio of 25∶1. Under the punishment condition, subjects received a moderate electric shock (titrated for each subject at level 5 on a 10-point scale with 1 indicating no feeling at all and 10 indicating a little painful but still tolerable, see below), but nothing for each correct response. Under both conditions, the same information feedback was also provided for 0.7 s, with a blue or red frame around the image to indicate correct and incorrect responses, respectively.

Subjects finished two reward reversal learning runs and two punishment reversal learning runs in two separate scan sessions that were approximately one week apart, with the order of task fully counterbalanced across subjects. Each run consisted of 10 mini-blocks of 4 images (two new images as learning trials and two old images from the last block as reversal trials) that were repeated 5 times. Specifically, each stimulus was reversed only once and was phased out of the experiment once the assigned repetitions were completed. The first block contained only 4 learning images, and only half of the images were included in the second block as reversal images. The contingency for the last 2 new images in the last mini-block was not reversed. To prevent subjects from being able to predict reversals, the old images might be repeated an additional 0,1, 2 or 3 times before the contingency was reversed. As a result, each image was repeated 5–8 times during acquisition and 5 times during reversal. We did not expect this manipulation to affect the difficulty in reversal learning as a previous study suggested that subjects learned the new contingency equally well for trials that were repeated 6 and 12 times during acquisition [41]. Overall, each run had a total of 218 trials with 22 learning images and 18 reversal images.

The trials were presented in mini-blocks to reduce working memory load (subjects only needed to keep 4 stimuli in mind at any given point in time), and also help to control inter-repetition interval (IRI) for each stimulus, a variable that has been shown to influence learning difficulty as well as retention of learning [46]. Trials within a mini-block were pseudo-randomized such that no stimulus repeated in succession. An event-related design was used in this fMRI study. The inter-trial-interval (ITI) was jittered between 2.5 to 7.8 seconds, and an in-house program was used to optimize design efficiency [47].

### Electric shock stimulator configuration and shock level determination procedure

We used a Grass SD9k square pulse stimulator (The Grass Technologies, http://www.grasstechnologies.com) to generate electric shocks. An MRI-safe electrode was attached to the subjects' left ankle. To determine the desirable level of stimulation for each subject, the voltage was initially set at 20 v and the subject was required to rate the level of pain on a 10-point scale, with 1 indicating no feeling at all and 10 indicating painful but tolerable. Based on subject's rating, the voltage was set to generate a pain level of 5.

### Functional imaging procedure

Subjects lay supine on the scanner bed, and viewed visual stimuli back-projected onto a screen through a mirror attached to the head coil. Foam pads were used to minimize head motion. Stimulus presentation and timing of all stimuli and response events were achieved using Matlab (Mathworks) and Psychtoolbox (www.psychtoolbox.org) on a MacBook Pro. Participants' responses were collected online using a MRI-compatible button box. An event-related design was used in this fMRI study.

fMRI imaging was conducted with a 3T Siemens MAGNETOM Tim/Trio scanner in the Dana and David Dornsife Cognitive Neuroscience Imaging Center at the University of Southern California. Functional scanning used a z-shim gradient echo EPI sequence with PACE (prospective acquisition correction). This specific sequence is designed to reduce signal loss in the prefrontal and orbitofrontal areas. The PACE option can help reduce the impact of head motion during data acquisition. The parameters are: TR = 2000 ms; TE = 25 ms; flip angle = 90°; 64×64 matrix size with a resolution of 3×3 mm^2^. Thirty-one 3.5-mm axial slices were used to cover the whole cerebrum and most of the cerebellum with no gap. The slices were tilted about 30 degrees clockwise from the AC–PC plane to obtain better signals in the orbitofrontal cortex. The anatomical T1-weighted structural scan was acquired using an MPRAGE sequence (TI = 800 ms; TR = 2530 ms; TE = 3.1 ms; flip angle 10; 208 sagittal slices; 256×256 matrix size with spatial resolution as 1×1×1 mm^3^).

### fMRI data preprocessing and statistical analysis

Image preprocessing and statistical analysis were carried out using FEAT (FMRI Expert Analysis Tool) version 5.98, part of the FSL package (FMRIB software library, version 4.1.8, www.fmrib.ox.ac.uk/fsl). The first four volumes before the task were automatically discarded by the scanner to allow for T1 equilibrium. The remaining images were then realigned to compensate for small residual head movements that were not captured by the PACE sequence [48]. Translational movement parameters never exceeded 1 voxel in any direction for any subject or session. The data were filtered in the temporal domain using a non-linear high pass filter with a 100 s cut-off, and spatially smoothed using a 5 mm full-width-half-maximum (FWHM) Gaussian kernel. A three-step registration procedure was used whereby EPI images were first registered to the matched-bandwidth high-resolution scan, then to the MPRAGE structural images, and finally into standard (MNI) space, using affine transformations [48]. Registration from MPRAGE structural images to standard space was further refined using FNIRT nonlinear registration [49]. Statistical analyses were performed in the native image space, with the statistical maps normalized to the standard space prior to higher-level analysis.

The data were modeled at the first level using a general linear model within FSL's FILM module. The experimental design allowed us to differentiate two components underlying reversal learning: (i) the detection of unexpected outcome, and (ii) the expression of new behavior under the interference of old behavior. For the first component, we compared the first error (1E) between reversal and acquisition. For the second component, we compared the correct trials during repetition 2 to 5 (2–5C) between reversal and acquisition. The first correct trial (1C), and all other error trials (2–5E) for acquisition and reversal were also separately modeled to examine the effects of reward and punishment processing. To control for the informational aspect of the feedback (e.g., correct vs. incorrect, and the requirement of behavioral change), regions sensitive to reward processing (i.e., money) were obtained by comparing all correct trials in the reward condition with those in the punishment condition. Similarly, regions sensitive to punishment processing (i.e., electric shock) were obtained by comparing all incorrect trials in the punishment condition with those in the reward condition. The trials for repetition 6 to 8 during acquisition were included as one covariate of no interest. The event onsets were convolved with a canonical hemodynamic response function (HRF, double-gamma) to generate the regressors used in the GLM. Temporal derivatives were included as covariates of no interest to improve statistical sensitivity.

A higher-level analysis was used to examine the effect of feedback (reward vs. punishment) on reversal learning by using a fixed effect model. These contrast results were then input into a random-effect model for group analysis using a FLAME (FMRIB's Local Analysis of Mixed Effects) stage 1 simple mixed effect model [50], [51], [52]. Group images were thresholded using cluster detection statistics, with a height threshold of *z*>2.3 and a cluster probability of *P*<0.05, corrected for whole-brain multiple comparisons using Gaussian Random Field Theory (GRFT).

### Conjunction analysis

To examine the overlapping mechanisms for reversal learning by reward and by punishment, conjunction analysis was performed to contrast acquisition and reversal, using the procedure suggested by Nichols et al. [53]. Accordingly, thresholded maps for each condition were binarized, and multiplied—thus revealing brain regions that were significantly activated in both conditions.

### Region-of-interest (ROI) analyses

ROIs were created by extracting the clusters showing common activation for reward and punishment reversal learning. As previous research has suggested a specific role of dorsal striatum in punishment learning [36], the bilateral caudate were anatomically defined according to the Oxford-Harvard Probability map included in the FSL package. Using these regions of interest, ROI analyses were performed by extracting parameter estimates (betas) of each event type from the fitted model and averaging across all voxels in the cluster for each subject. Percent signal changes were calculated using the following formula: [contrast image/(mean of run)]×ppheight×100%, where ppheight is the peak height of the hemodynamic response versus the baseline level of activity [54].

## Results

### Behavioral results

**Figure 2:**
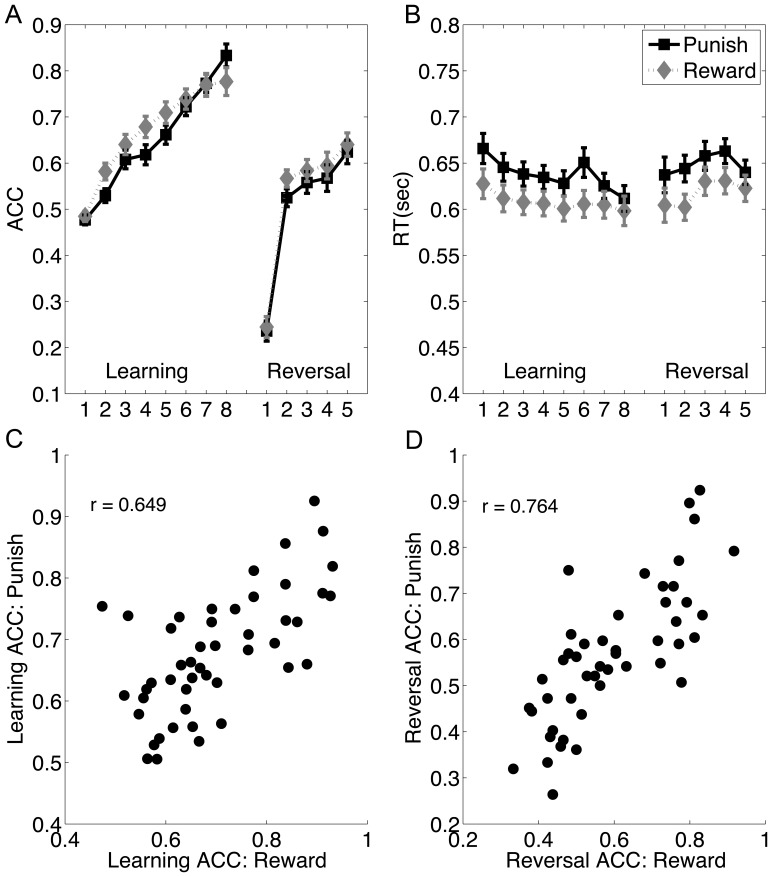
Behavioral results. The averaged accuracy (A) and reaction time (B) for each repetition during initial acquisition and reversal were plotted, separately for reward and punishment conditions. Error bars indicate one standardized error (SE). The scatter plots show the correlation of performance (accuracy) between reward and punishment during initial acquisition (C) and subsequent reversal learning (D).

Figure 2 shows the behavioral data during acquisition and reversal for both reward and punishment condition. For accuracy, repeated measure analysis of variance (ANOVA) on repetition 2 to 5 revealed a significant effect of repetition (F(3,46) = 48.33, p<.0001). Learning under reward was better than under punishment (F(1,46) = 15.43, p = .0003), but there was no significant repetition by feedback interaction (F(3,46) = 0.83, p = .48). For reaction time, we found that learning significantly increased the speed (F(3,46) = 2.80, p = .04), and it took longer to make decisions under punishment than under reward (F(1,46) = 6.04, p = .018). But again there was no significant repetition by feedback interaction (F(3,46) = 0.27, p = .85).

Upon the first reversal trial, the accuracy dropped to 24.5% and 23.6% in the reward and punishment conditions respectively (t(46) = 0.43, p = .66), suggesting that our manipulation successfully prevented the prediction of reversal. However, subjects could quickly reconfigure the stimulus-reward mapping on the second post-reversal trial [accuracy: 56.7% and 52.5% in the reward and punishment conditions, respectively, t(46) = 2.03, p = .048]. From rep 2 to rep 5 post-reversal, the accuracy continued to improve (F(3,46) = 13.28, p<.0001) and the RT initially increased from rep 2 to 4 (F(2,46) = 11.98, p<.0001) and then decreased from rep 4 to 5(F(1,46) = 13.53, p<.0001). Accuracy was marginally better for reversal learning under reward than punishment (F(1,46) = 3.32, p = .075), and the RT was longer under punishment (F(1,46) = 5.81, p = .02). The interaction between learning type (reward vs. punishment) and repetitions (rep 2 to 5) was marginally significant for RT (f(3,138) = 2.38, p = 0.07), but not significant for accuracy (f(3,138) = 0.58, p = .62).

Compared to initial acquisition, a 3-way ANOVA suggested that the accuracy from rep 2 to rep 5 during reversal was lower (F(3,46) = 29.62, p<.0001) and the RT was longer (F(3,46) = 34.93, p<.001), suggesting that there were strong cognitive costs when expressing the new behaviors under the interference of old prepotent responses. There was also a significant reversal by repetition interaction in accuracy (F(3,138) = 5.29, p = .0017) and RT (F(3,138) = 9.59, p<.0001), indicating that the performance improved at a slower rate under reversal than under acquisition. There was a marginally significant feedback type by reversal interaction in accuracy (F(1,46) = 3.78, p = .06), but not in RT (F(1,46) = .006, p = .94), suggesting the accuracy advantage with reward feedback was reduced during reversal. The feedback type by repetition interactions or the three-way interactions were not significant (all ps>.1).

There were strong correlations in accuracy between reward and punishment condition during both initial acquisition (r = .65, p<.0001) (Figure 2C) and reversal (r = .76, p<.0001) (Figure 2D).

Taken together, the behavioral results suggest that it took more effort to learn the reversed contingency than to learn the initial association. The reversal effect was larger for reward than for punishment learning, probably due to the better initial acquisition performance under the reward condition. However, there was no interaction between feedback type and reversal effect in RT, enabling us to compare the reversal effect between the two conditions without being confounded by RT.

### fMRI Results

#### Brain regions involved in detecting contingency changes

To examine the neural mechanisms for detecting contingency change, we compared the first incorrect reversal trials with the first incorrect acquisition trials. Unlike previous studies that compared incorrect reversal trials with correct acquisition trials, this contrast is not confounded by response accuracy.

**Figure 3:**
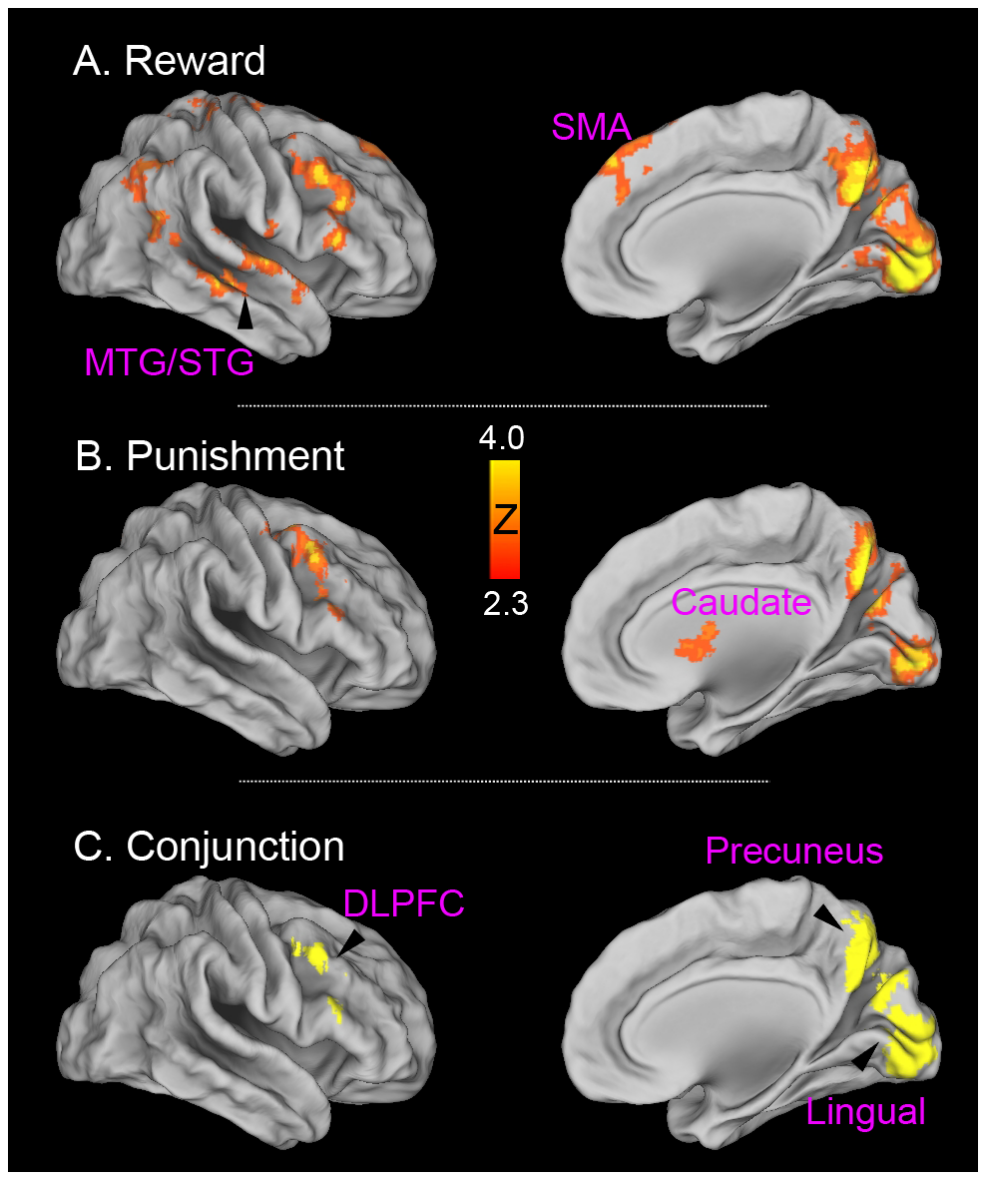
Brain regions associated with contingency change detection (1^st^ reversal error >1^st^ acquisition error). Significant activation for reward (A), punishment (B) and their conjunction (C), are rendered onto a population-averaged surface atlas using multi-fiducial mapping[88]. All activations were thresholded by using cluster detection statistics, with a height threshold of z>2.3 and a cluster probability of P<0.05, corrected for whole-brain multiple comparisons.

In the reward condition, we found strong activation in the right dorsolateral frontal cortex (DLPFC) (MNI: 52, 26, 28, Z = 4.94) and precuneus (MNI: 0, −60, 44; Z = 4.67) that included the cuneus and extended all the way down to the lingual gyrus (MNI: 8, −92, −2; Z = 6.02) (Figure 3A). The left (MNI: −56, −2, −2, Z = 4.22) and right (MNI: 66, −6, 2, Z = 4.27) middle/superior temporal gyrus, and the middle portion of the superior frontal gyrus (MNI: 4, 48, 44; Z = 4.13) were also found in this contrast. In the punishment condition, the peak of activation was found in the right IFG (MNI: 44, 10, 50, Z = 4.4) and precuneus (MNI: 2, −64, 34, Z = 5.52) that also included the cuneus and extended down to the lingual gyrus (MNI: 6, −90, −2; Z = 5.41), as well as in the left (MNI: −8, 8,10; Z = 3.46) and right caudate (MNI: 10,2,12; Z = 4.36) (Figure 3B). Direct comparison revealed no differences between the two conditions. Because previous research suggested a specific role of the dorsal striatum in punishment learning [36], anatomical ROIs were defined to examine whether there were subtle differences between reward and punishment reversal learning (Figure S1). Only a small trend of feedback type by reversal interaction were found in the left (F(1,46) = 2.70, p = .10) and the right (F(1,46) = 2.98, p = .09) caudate, providing weak evidence for the specificity of caudate in punishment reversal learning.

A conjunction analysis was conducted to formally examine overlapping mechanisms in detecting contingency changes under both conditions. This analysis revealed common activations in the right DLPFC (center of gravity [COG]: MNI: 48, 18, 36), and the precuneus/lingual gyrus (COG: 4, −76, 22).

The reversed contrast (first acquisition errors > first reversal errors) revealed activations in the bilateral visual cortex for both conditions (Figure S2), which may be related to repetition priming of visual object processing, as the images were novel during the first acquisition but were studied 5 to 8 times before reversal. Stronger activation was also found in the default network, including the ventral medial prefrontal cortex (VMPFC), and in the punishment condition only, the posterior cingulate cortex (PCC) and the lateral region of the superior frontal gyrus (SFG) [55], [56], which might be related to decreased processing demand. These activations will not be discussed further.

#### Brain regions involved in inhibiting old contingency and expressing new behavior

In a second contrast, we compared all the correct trials during reversal with those during acquisition. Behavioral results suggested worse performance during reversal than under initial learning even after 5 repetitions, indicating extended reversal costs, and therefore this comparison could reveal neural regions involved in overcoming the old contingency and expressing the new behavior under interference. Again, since we only compared the correct trials, our results will not be confounded by response accuracy.

**Figure 4:**
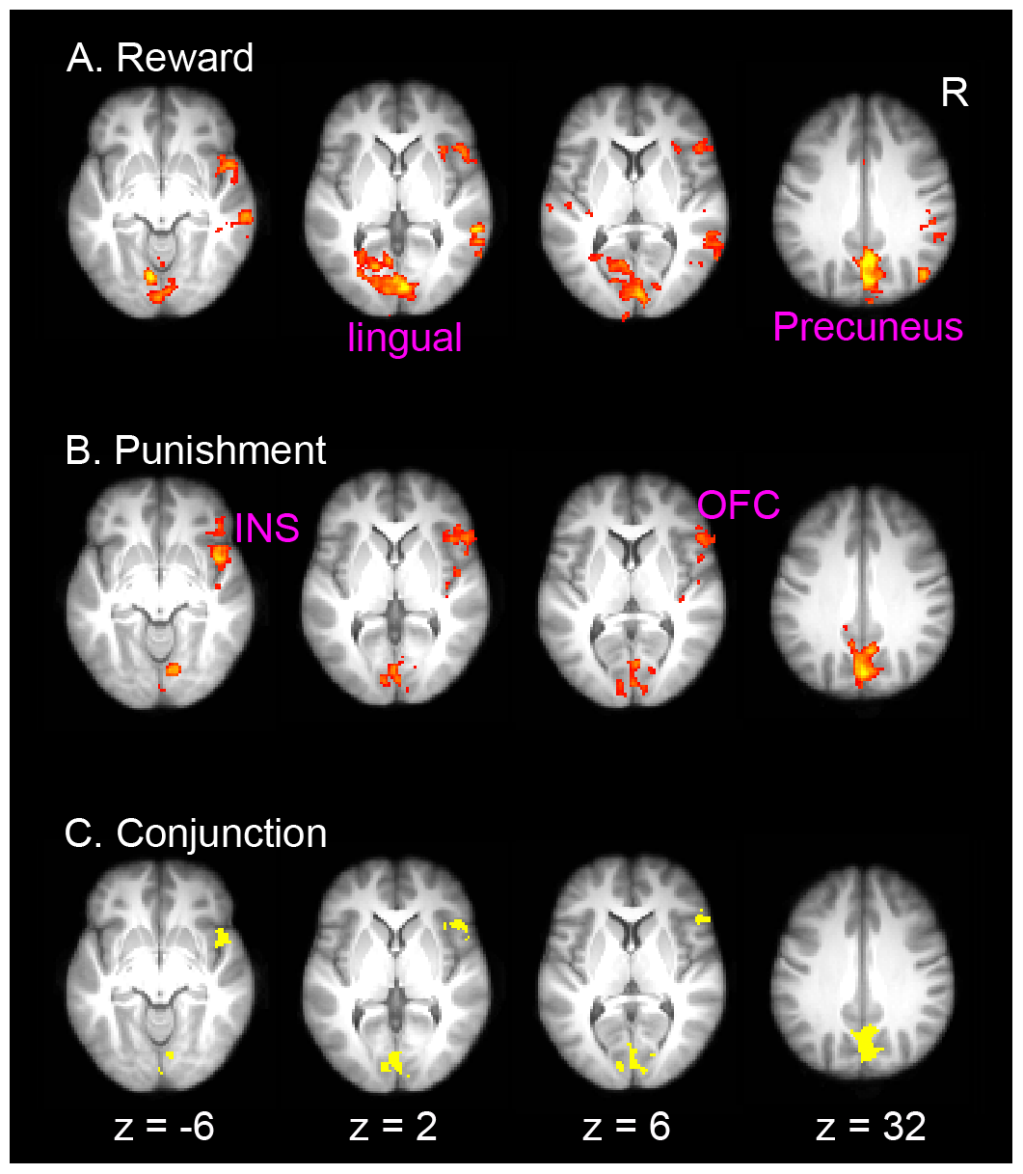
Brain activation associated with the inhibition of old contingency and the expression of new behaviors (correct reversal > correct acquisition). Significant activation for reward (A), punishment (B) and their conjunction (C), are overlaid on axial slices of the group mean structural image. All activations were thresholded by using cluster detection statistics, with a height threshold of z>2.3 and a cluster probability of P<0.05, corrected for whole-brain multiple comparisons.

In the reward condition, we found significantly stronger activations in the right lateral OFC and adjacent insula (MNI: 50, 22, 4; Z = 3.31), the precuneus (MNI: 2, −74, 42; Z = 4.82), the lingual gyrus (MNI: −8, −74, −10; Z = 4.61) and the supplementary motor cortex (MNI: 0, −2, 52; Z = 3.35). Other regions included the left superior temporal gyrus (MNI: −62, −24, 12; Z = 3.30), the right middle temporal gyrus (MNI: 52, −12, −18; Z = 3.85), the right lateral occipital cortex (MNI: 44, −72, 28; Z = 4.25), and the right cerebellum (MNI: 14, −54, −24; Z = 4.05) (Figure 4A). In the punishment condition, there were stronger activations in the right lateral OFC/insula (MNI: 46, 8, −8; Z = 3.77), precuneus (MNI: 4, −68, 42; Z = 4.5) and the lingual gyrus (MNI: 12, −78, −6; Z = 3.69) (Figure 4B). Conjunction analysis revealed overlapping activations in the right lateral OFC/insula (COG in MNI: 48, 16, −2), the precuneus (COG in MNI: 2, −68, 38) and the lingual gyrus (COG in MNI: 2, −84, 0). No significant differences were found between the two conditions.

The reversed contrast (Learning > Reversal) revealed strong activities in the bilateral ventral visual cortex (Figure S3), which again may be related to the differences in the familiarity of the visual objects.

### ROI analysis: Functional dissociation of DLPFC and lateral OFC

**Figure 5:**
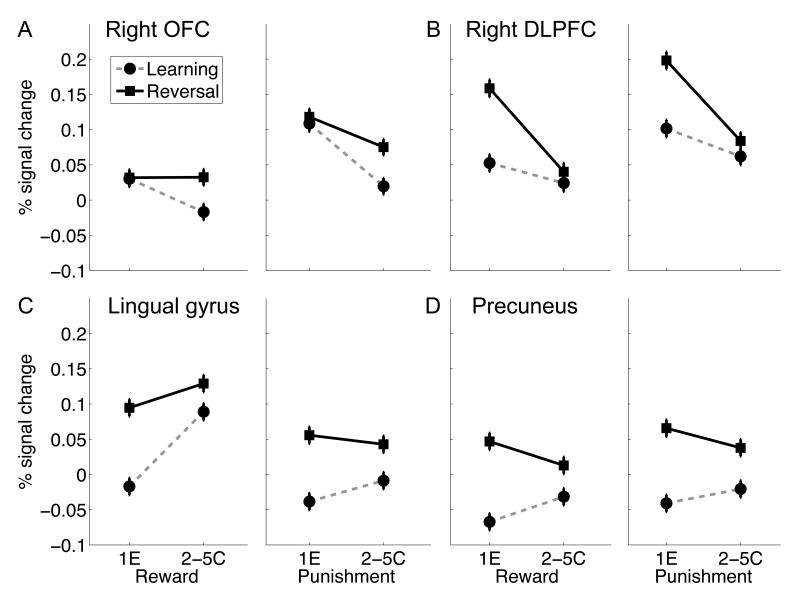
ROI results. Percentage signal change in the right OFC (A), the right DLPFC (B), the lingual gyrus (C) and the precuneus (D), is plotted as a function of learning stage (1E vs. 2–5C), learning condition (reward vs. punishment) and reversal (learning vs. reversal). Error bars indicated with-subject standard error. 1E: first error; 2–5C: correct trials during repetitions 2 to 5.

To further examine whether the DLPFC and lateral OFC were each specifically involved in one process but not the other, we performed additional ROI analysis to examine process (first error vs. correct behavior expression) by reversal interaction (Figure 5). Three-way (with feedback type, reward vs. punishment, as an additional factor) repeated ANOVA revealed significant process (1E vs. 2–5C) by reversal interaction for both the right DLPFC (F(1,46) = 28.63; p<.0001) and the lateral OFC (F (1,46) = 8.36, p = .0058), indicating that the right DLPFC was involved in contingency change detection whereas the lateral OFC was involved in inhibiting old associations. Significant process by reversal interactions were also found in the precuneus (F(1,46) = 9.38, p = .004) and the lingual gyrus (F(1,46) = 10.68, p = .002), indicating they were more heavily involved in detecting contingency changes.

To further show that the lateral OFC activation was not simply driven by the first one or two repetitions during reversal learning, we plotted the lateral OFC activation across the whole period of acquisition and reversal, separately for each repetition (Figure S4). Focusing on repetition 2 to 5, we found no repetition by reversal interaction in either the reward condition (F(3,138) = 0.35, p = .79) or the punishment condition (F(3,138) = 1.70, p = .17). For both conditions, a strong reversal effect was still present on the fifth repetition (ps<.018).

### The right DLPFC and lateral OFC were also sensitive to punishment

**Figure 6:**
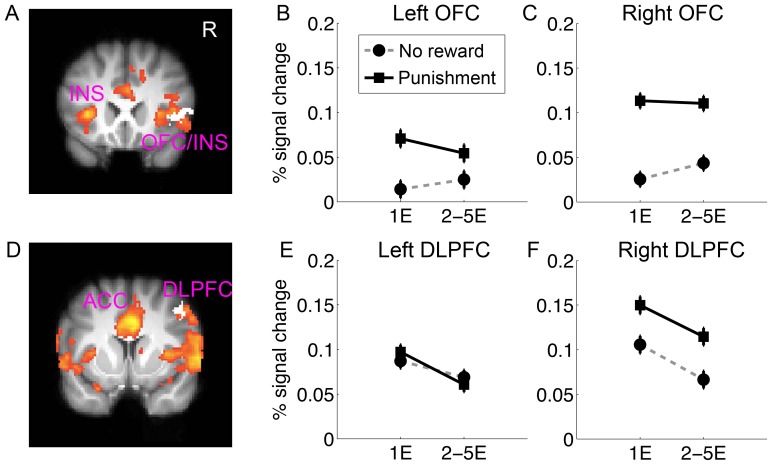
Neural response to punishment compared to no reward. Significant activation for in the lateral OFC and insula (A), the DLPFC (D), as well as in ACC and bilateral insula, are overlaid on coronal slices of the group mean structural image. All activations were thresholded by using cluster detection statistics, with a height threshold of z>2.3 and a cluster probability of P<0.05, corrected for whole-brain multiple comparisons. The right OFC and right DLPFC ROIs showing common reversal effect for reward and punishment are shown in white color, on panel A and D respectively. Their left homologues were defined by a left-right flipping. Percentage signal change in the left (B) and right (C) OFC, the left (E) and right (F) DLPFC, is plotted as a function of learning stage (1E vs. 2–5E), feedback type (no reward vs. punishment). Error bars indicated with-subject standard error. 1E: first error; 2–5E: error trials during repetitions 2 to 5.

Finally, our design allowed us to examine the neural response to reward and punishment, without being confounded by the information/processes associated with them, such as correct/incorrect, the occurrence of contingency change or the requirement for maintaining or switching responses. To examine the neural mechanisms associated with punishment processing, we compared all the incorrect trials during both acquisition and reversal under punishment (where subjects received a mild shock) with those under reward (where subjects received nothing). This analysis revealed strong responses in the bilateral insula and the anterior cingulate cortex that are associated with somatosensory processing and also the affective aspects of pain (Figure 6A & D). Interestingly, we also found strong activation in the right dorsolateral prefrontal cortex and the lateral orbital frontal cortex, but no above-threshold activation in the left homologues. No significant effect of reward processing was found, probably due to the small reward used in this study.

To formally examine whether the same regions implicated in reversal learning (i.e., right DLPFC and lateral OFC) also showed sensitivity to punishment, we took these ROIs and their left hemisphere homologues, and examined their responses to punishment. We found that both the left (F(1,46) = 5.72, p = .02) and right OFC (F(1,46) = 13.14, p<.001) showed sensitivity to punishment (Figure 6 B & C). However, the effect was much stronger in the right hemisphere as indicated by the hemisphere by condition (reward vs. punishment) interaction (F(1,46) = 4.57, p = .038). Similarly, the right DLPFC (F(1,46) = 6.83, p = .01) but not the left DLPFC (F(1,46) = 0.003, p = .95) showed sensitivity to punishment (Figure 6 E & F), and this laterality was also confirmed by hemisphere by condition interaction (F(1,46) = 8.25, p = .006).

## Discussion

The present study revealed distinct neural networks that contributed to different aspects of reversal learning, and how they were modulated by reward and punishment. This design also enabled us to clearly dissociate processes associated with feedback processing and reversal learning. We found that the dorsal lateral prefrontal cortex was strongly activated when receiving unexpected negative feedback, whereas the right lateral OFC was involved in inhibiting the old contingency and the expression of new behavior. Importantly, we found that this pattern was highly consistent across different types of feedback, i.e., monetary reward and mild electric shock. In addition, these two regions on the right side were also particularly sensitive to punishment, more so than their left homologues. These results provide new insight into the neural mechanisms of reversal learning, and can be leveraged to understand risky behaviors in vulnerable populations.

Consistent with previous studies[29], [41], we found that the right DLPFC showed elevated activation when the subject received unexpected negative feedback during reversal, as compared to that during acquisition. Compared to the negative feedback during the initial guessing stage of learning when no contingency was established, the negative feedback during reversal signals the change of contingency and the requirement to switch response in subsequent learning. Our results further suggest that the DLPFC activity, in response to contingency change, could be triggered either by the absence of an expected reward (i.e., reward condition) or the presence of an unexpected electric shock (i.e., punishment condition). It should be emphasized that by comparing the post-reversal errors with initial guessing errors, this contrast effectively subtracted out the activation due to the processing of specific forms of feedback.

The region of DLPFC identified in this study is located very closely to that found in Ghahremani et al. [41], but is more dorsal than the inferior frontal gyrus involved in response inhibition [57], [58]. Indeed, conjunction analysis between reversal learning and response inhibition showed non-overlapping activation in the DLPFC region [41]. This result is compatible with the suggestion that the DLPFC is involved in generalized contingency learning, like the detection of contingency change that is either value-relevant or value-irrelevant [59]. Others suggest that the DLPFC is involved in attention shift [13] or plays a higher-level role in attentional control [26]. This general role of attentional control would suggest an enhanced activity when the contingency was changed. As suggested by Mitchell et al. [29], although this attentional control might not be crucial for simple object reversals where the demands on attention are relatively low [13], it is important when multiple stimuli and stimulus properties are involved and only the contingency for some of the stimuli were changed. Future lesion and virtual lesion studies are required to test whether the DLPFC is necessary for reversal learning under this circumstance.

On the other hand, the comparison between the post-reversal correct trials with the correct acquisition trials revealed strong activity in the right lateral OFC. The critical process in this contrast is that during acquisition, no strong prepotent responses are established, whereas reversal learning poses strong requirements to inhibit the old contingency and to express the new behavior under interference. This finding is consistent with many previous observations implicating its role in behavioral flexibility by reversing established stimulus-response contingency [20], [24], [41], [60], [61], [62].

Whether the lateral OFC is involved in value representation or behavioral control has been extensively debated [63]. By using a design that enabled us to disentangle the feedback process, response reversal, reward, and punishment, we provide solid evidence that the lateral OFC was involved in both behavioral control AND punishment processing. The lateral OFC activation extended to the anterior insula, which has been considered an extension of the frontal operculum [64]. Our finding is consistent with the hypothesis that the lateral OFC and anterior insula are a part of the saliency and behavioral control network [65], [66], which play a general role in transforming interoceptive signals to motivational behaviors [67], [68]. As punishing feedback in both the learning and reversal stage signals the necessity for response switch, it is not surprising that we found equally strong lateral OFC-insula activation for the first reversal error and the first acquisition error (Figure 5). The strong right lateralization is consistent with the asymmetry in peripheral autonomic efferents and homeostatic afferents, with the right hemisphere more involved in sympathetic response and the left hemisphere in parasympathetic response [69]. Also consistent with this view, this area is involved in inhibiting both manual and vocal responses driving by a rare stop signal [70]. In probabilistic reversal learning, this area is activated by punishing feedback preceding a switch [71], indicating its role in transferring the punishing signal to behavioral change.

The common lateral OFC activations for both reward and punishment reversal learning extends previous studies on reversal learning and provides clear evidence for a common role in behavioral flexibility driven by the absence of predicted reward and the presence of unpredicted punishment. This result is consistent with a recent study, which found similar lateral OFC activation for reversals involving positive and negative associations. Both were stronger than for that involving neutral associations [72]. Previous studies on reinforcement learning have found distinct neural mechanisms for learning by reward and that by punishment [36], [37], [39], [73]. In particular, although several studies on reward and punishment reinforcement learning also used a reversal learning paradigm [37], [73], these studies focused on prediction error and the dopamine system in the striatum, which is very different from the way we analyzed the data. Similarly, although lesion studies have suggested that the ventromedial PFC was responsible for reversal learning [17], [74], especially that driven by negative feedback [35], these studies did not directly compare reversal learning by reward and punishment. Indeed, in the Iowa gambling task (IGT) that involves reversal learning (albeit more complex), the VMPFC patients were impaired in both the original task (decks ABCD, where some decks are rewarded first and then punished) and the variant IGT (decks EFGH where some decks are punished and then rewarded [75].

Our results are in general agreement with a previous study using a similar paradigm [41]. One difference is that we found right OFC activation differences between acquisition and reversal on repetition 3–5 whereas they did not. We think this discrepancy might be mainly caused by the differences in behavioral performance. In their study, the accuracy during reversal was high (around 75% on second post-reversal trial and 90% on the 5^th^ post-reversal), whereas, in this study, the accuracy on the second post-reversal is 57% and 53%, and 64% and 62% on the 5^th^ repetition, for reward and punishment respectively. Compared to the acquisition stage, behavioral performance during reversal learning was worse after 5 repetitions, as indicated by the lower accuracy and longer RT. The lower performance during reversal would require more extended involvement of behavioral control [43].

Another factor that might contribute to this discrepancy might be the difference in subject population. As a part of a large study on risk behaviors in MSM, this study only recruited MSM as subjects. Although we are not aware of findings of major differences between MSM and heterosexual subjects, significant gender differences have been found in the metabolism [76] and activation [77] of the OFC, as well as in behaviors associated with OFC function, such as the Iowa gambling task [78], [79]. The consequences of lateral OFC lesions on behaviors are also modulated by gender [71]. In addition, females have increased DA synthesis relative to males [80] and reduction of global DA synthesis results in significantly improved punishment reversal learning in female but not male subjects [37]. Given these significant gender differences, future studies definitely need to directly compare reversal learning between males and females with functional imaging. Still, direct comparison between MSM and heterosexual subjects with functional imaging methods are also warranted.

Finally, based on previous results showing that there are overlapping neural mechanisms for reward and reward prediction errors for primary rewards using juice and secondary rewards using money 44,45, the present study chose monetary reward as it is much easier to implement and by far the most frequently used reward with humans. This, however, introduced a comparison between a primary reinforcer (shock) for punishment, and a secondary reinforcer (money) for reward. Further, it is difficult to determine whether the two reinforcers are equivalent in terms of magnitude. This design did not contaminate our analysis of the reversal effect, as we directly contrasted reversal learning with initial acquisition, and any differences between reward and punishment learning would have cancelled out. Nevertheless, it might have contributed to the differences between reward and punishment processing, both at the behavioral level and neural level. For example, our behavioral data suggested that subjects learned faster by reward than by punishment. At the neural level, the right hemisphere was more involved in punishment than was the left hemisphere, whereas no significant activation was found for reward. Previous studies have suggested that reward and punishment are represented differently in the orbitofrontal cortex. In particular, punishment has been found to be lateralized to the right OFC [19], [81], whereas reward has been found to activate the medial OFC (also called ventromedial PFC)[19], [82], [83], [84] or the left OFC [81], [84], [85]. Our study provides a way to separate reward/punishment processing, the informational aspect of the feedback and the requirement for behavioral control, and the results are partially consistent with previous observations. Further studies should be conducted to examine these effect using primary reward (such as juice and arousal pictures) and punishment with comparable magnitude.

To conclude, using an effective design to contrast initial acquisition with reversal learning, and to contrast reward and punishment feedback while matching the requirement on behavioral control, our study provides clear insight into the neural mechanisms of reversal learning. Whereas the right lateral OFC and anterior insula are involved in transforming affective (especially negative) feedback to behavioral adjustment, the DLPFC is particularly activated when such feedback signals a change in contingency and thus a higher level of attention control is warranted. Importantly, we show that these mechanisms can be effectively triggered by the delivery of unexpected punishment or the withdrawal of expected reward, offering strong redundancy and flexibility to the human behavioral control system. This study also presents a first step to understanding the behavioral flexibility mechanisms in the MSM population, which has been shown to be vulnerable to risky behavior, such as risky sex and HIV [86], [87]. Future studies need to examine how these mechanisms can help us understand risky behaviors in this population.

## Supporting Information

Figure S1 **ROI results of the bilateral caudate.** The bilateral caudate were anatomically defined according to the Oxford-Harvard Probability map included in the FSL package. Percentage signal change in the left (A) and the right caudate (B), is plotted as a function of learning stage (1E vs. 2–5C), learning condition (reward vs. punishment) and reversal (learning vs. reversal). Error bars indicate with-subject standard error. 1E: first error; 2–5C: correct trials during repetitions 2 to 5. Repeated measure ANOVA revealed only a small trend of feedback type by reversal interaction in the left (F(1,46) = 2.70, p = .10) and the right (F(1,46) = 2.98, p = .09) caudate, providing weak evidence for the specificity of caudate in punishment reversal learning.
(TIF)

Figure S2 **Brain regions associated stronger activation for first acquisition error than for first reversal error.** Significant activations for reward (A), punishment (B), are rendered onto a population-averaged surface atlas using multi-fiducial mapping (Van Essen, 2005). All activations were thresholded by using cluster detection statistics, with a height threshold of z>2.3 and a cluster probability of P<0.05, corrected for whole-brain multiple comparisons. Strong activations were found in the bilateral visual cortex for both conditions, as well as in the default network, which may be related to the repetition priming of visual object processing, and less processing requirement.
(TIF)

Figure S3 **Brain regions associated stronger activation for correct acquisition trials than for correct reversal trials (repetitions 2 to 5).** Significant activations for reward (A), punishment (B), are rendered onto a population-averaged surface atlas using multi-fiducial mapping (Van Essen, 2005). All activations were thresholded by using cluster detection statistics, with a height threshold of z>2.3 and a cluster probability of P<0.05, corrected for whole-brain multiple comparisons. Strong activations were again found in the bilateral visual cortex for both conditions, related to the repetition priming of visual object processing.
(TIF)

Figure S4 **ROI results of the lateral OFC.** Error bars indicate with-subject standard error. 1E: first error; 2–5C: correct trials during repetitions 2 to 5. This analysis showed consistent rOFC activation during all repetitions of reversal, as indicated by the lack of repetition by reversal interaction under either reward (F(3,138) = 0.35, p = .79) or punishment condition (F(3,138) = 1.70, p = .17).
(TIF)
