# Supplementary figures and images for: Common Neural Mechanisms Underlying Reversal Learning by Reward and Punishment
Source: PLoS One. 2013 Dec 11;8(12):e82169. doi: 10.1371/journal.pone.0082169 (PMC3859585; doi:10.1371/journal.pone.0082169)

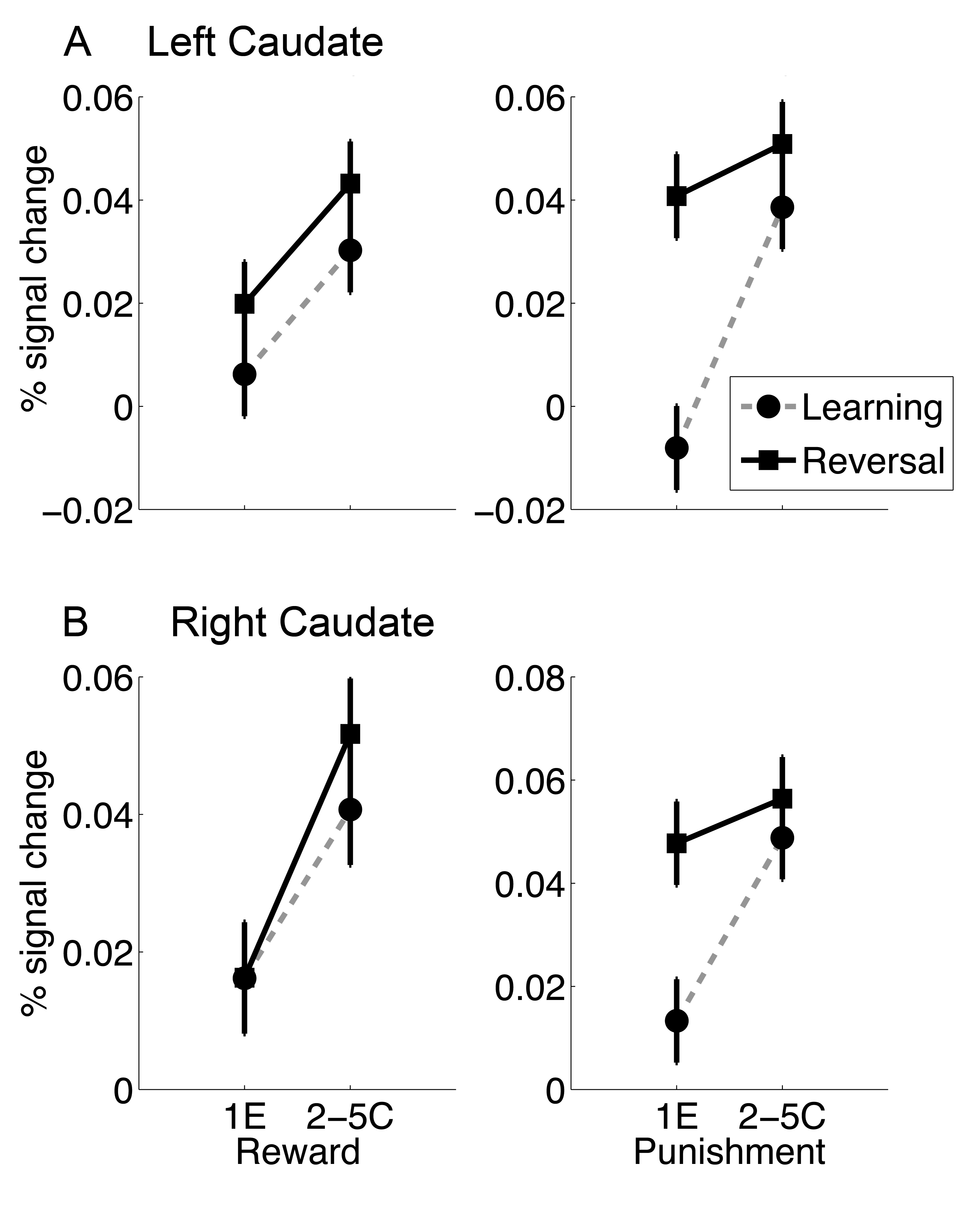

Supplement: Figure S1 — ROI results of the bilateral caudate. The bilateral caudate were anatomically defined according to the Oxford-Harvard Probability map included in the FSL package. Percentage signal change in the left (A) and the right caudate (B), is plotted as a function of learning stage (1E vs. 2–5C), learning condition (reward vs. punishment) and reversal (learning vs. reversal). Error bars indicate with-subject standard error. 1E: first error; 2–5C: correct trials during repetitions 2 to 5. Repeated measure ANOVA revealed only a small trend of feedback type by reversal interaction in the left (F(1,46) = 2.70, p = .10) and the right (F(1,46) = 2.98, p = .09) caudate, providing weak evidence for the specificity of caudate in punishment reversal learning. (TIF) [file pone.0082169.s001.tif]

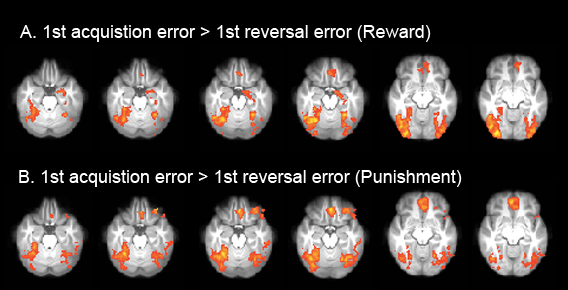

Supplement: Figure S2 — Brain regions associated stronger activation for first acquisition error than for first reversal error. Significant activations for reward (A), punishment (B), are rendered onto a population-averaged surface atlas using multi-fiducial mapping (Van Essen, 2005). All activations were thresholded by using cluster detection statistics, with a height threshold of z>2.3 and a cluster probability of P<0.05, corrected for whole-brain multiple comparisons. Strong activations were found in the bilateral visual cortex for both conditions, as well as in the default network, which may be related to the repetition priming of visual object processing, and less processing requirement. (TIF) [file pone.0082169.s002.tif]

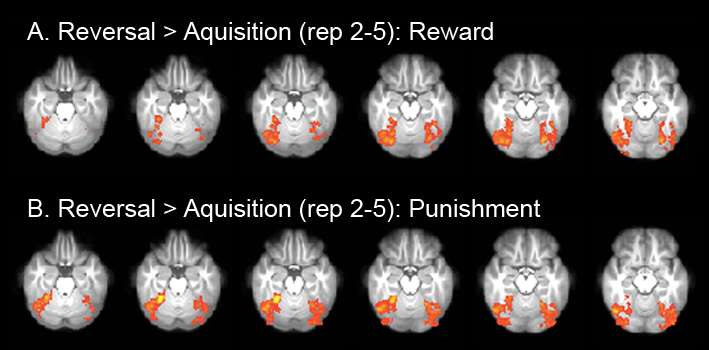

Supplement: Figure S3 — Brain regions associated stronger activation for correct acquisition trials than for correct reversal trials (repetitions 2 to 5). Significant activations for reward (A), punishment (B), are rendered onto a population-averaged surface atlas using multi-fiducial mapping (Van Essen, 2005). All activations were thresholded by using cluster detection statistics, with a height threshold of z>2.3 and a cluster probability of P<0.05, corrected for whole-brain multiple comparisons. Strong activations were again found in the bilateral visual cortex for both conditions, related to the repetition priming of visual object processing. (TIF) [file pone.0082169.s003.tif]

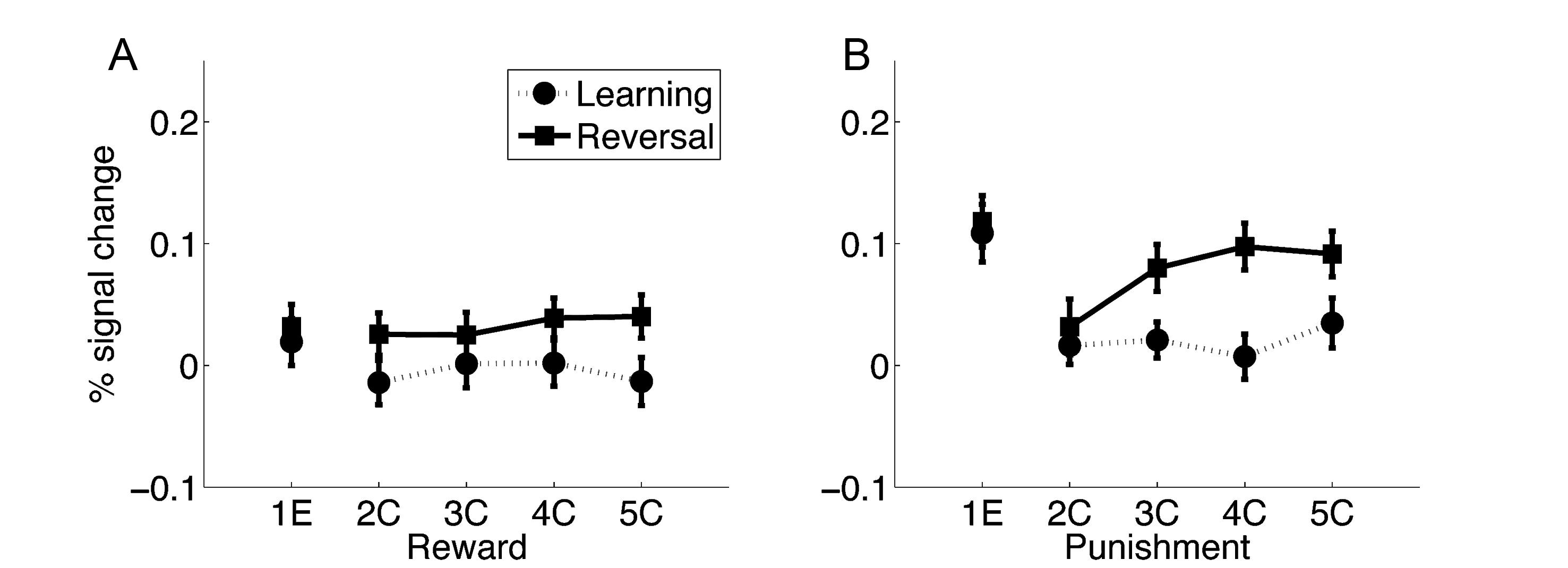

Supplement: Figure S4 — ROI results of the lateral OFC. Error bars indicate with-subject standard error. 1E: first error; 2–5C: correct trials during repetitions 2 to 5. This analysis showed consistent rOFC activation during all repetitions of reversal, as indicated by the lack of repetition by reversal interaction under either reward (F(3,138) = 0.35, p = .79) or punishment condition (F(3,138) = 1.70, p = .17). (TIF) [file pone.0082169.s004.tif]
